# The Association Between Lumbar Disc Degeneration and Paraspinal Muscle Morphology: The Role of Age in a Quantitative Magnetic Resonance Imaging-Based Study

**DOI:** 10.3390/biomedicines14081673

**Published:** 2026-07-25

**Authors:** Abdurrahim Tekin, Umut Çelik, Akın Öztürk, Salih Dere, Engin Can, Evren Sönmez, Lokman Ayhan, Suna Dilbaz, Nuri Serdar Baş, Serdar Çevik

**Affiliations:** Department of Neurosurgery, Kanuni Sultan Süleyman Training and Research Hospital, Atakent Mahallesi, Turgut Özal Bulvarı No: 46/1, Küçükçekmece, 34303 Istanbul, Türkiye; umutcelik3796@gmail.com (U.Ç.); akozturk@gmail.com (A.Ö.); salidere@hotmail.com (S.D.); ecan281@gmail.com (E.C.); evren-sonmez@hotmail.com (E.S.); op.dr.lokmanayhan@gmail.com (L.A.); sunadilbaz@gmail.com (S.D.); nuriserdarbas@gmail.com (N.S.B.); drserdarcevik@gmail.com (S.Ç.)

**Keywords:** lumbar disc degeneration, Pfirrmann score, multifidus, erector spinae, paraspinal muscles, fatty infiltration, magnetic resonance imaging

## Abstract

**Aim:** The relationship between lumbar intervertebral disc degeneration and paraspinal muscle degeneration remains controversial. Although previous studies have reported associations between disc degeneration and muscle atrophy and fatty infiltration, the effect of age on this relationship has not been sufficiently clarified. This study aimed to investigate the associations that cumulative and L4–L5 level-specific disc degeneration have with paraspinal muscle morphology, and to determine whether these associations persisted after adjustment for age. **Materials and Methods:** This retrospective cross-sectional study included 84 patients who underwent lumbar magnetic resonance imaging (MRI) for low back pain. Disc degeneration was evaluated at all lumbar levels according to the Pfirrmann classification, and the Total Pfirrmann Score was calculated. Relative functional cross-sectional area (rFCSA) and fatty infiltration (FI) measurements of the multifidus, erector spinae, and psoas muscles at the L4–L5 level were performed using ImageJ software. The relationships between variables were examined using Spearman correlation analysis. Multiple linear regression analysis was performed to investigate independent predictors. Age-controlled partial correlation analyses were also performed to evaluate the potential confounding effect of age. **Results:** Of the 84 patients included in the study, 48 (57.1%) were female and 36 (42.9%) were male, with a mean age of 42.3 ± 8.4 years. The mean Total Pfirrmann Score was 14.3 ± 3.8. A moderate positive correlation was found between age and the Total Pfirrmann Score (rho = 0.519; *p* < 0.001). Significant positive correlations were observed between the Total Pfirrmann Score and multifidus fatty infiltration (rho = 0.316; *p* = 0.003) and erector spinae fatty infiltration (rho = 0.300; *p* = 0.006). A weak negative correlation was detected between psoas rFCSA and the Total Pfirrmann Score (rho = −0.247; *p* = 0.023). Multifidus and erector spinae rFCSA were not significantly associated with the Total Pfirrmann Score (*p* = 0.094 and *p* = 0.095, respectively). At the L4–L5 level, higher Pfirrmann grade was associated with lower multifidus rFCSA (rho = −0.233; *p* = 0.033) and lower psoas rFCSA (rho = −0.319; *p* = 0.003), whereas the inverse association with erector spinae rFCSA was borderline (rho = −0.215; *p* = 0.050). Multiple linear regression analysis showed that age was the only statistically significant predictor of the Total Pfirrmann Score in the multivariable model (*p* < 0.001). After adjustment for age, the associations of multifidus FI (r = 0.193; *p* = 0.079) and erector spinae FI (r = 0.194; *p* = 0.078) with the Total Pfirrmann Score did not reach statistical significance. In the age-adjusted L4–L5 analysis, only the inverse association between Pfirrmann grade and psoas rFCSA remained statistically significant (r = −0.232; *p* = 0.034). **Conclusions:** Greater cumulative lumbar disc degeneration was associated with increased fatty infiltration of the multifidus and erector spinae muscles and lower psoas rFCSA, whereas multifidus and erector spinae rFCSA were not associated with the Total Pfirrmann Score. At L4–L5, greater disc degeneration was associated with lower multifidus and psoas rFCSA, while the association with erector spinae rFCSA was borderline. After adjustment for age, the associations of multifidus and erector spinae fatty infiltration with cumulative disc degeneration were attenuated and did not reach statistical significance; however, a weak independent association cannot be excluded.

## 1. Introduction

Low back pain is one of the most common musculoskeletal disorders worldwide and causes substantial loss of workforce and reduced quality of life [1]. Lumbar intervertebral disc degeneration (LIDD) is considered one of the most common structural causes of chronic low back pain. Magnetic resonance imaging is the most widely used method for evaluating the degenerative process, and the Pfirrmann classification is currently one of the most commonly used and best-validated systems for grading disc degeneration [2].

The Pfirrmann classification enables objective grading of intervertebral disc degeneration by evaluating morphological features such as disc signal intensity, the distinction between the nucleus pulposus and annulus fibrosus, disc structure, and disc height [2]. In recent years, the use of the total Pfirrmann score, which reflects the degenerative burden of the entire lumbar spine rather than a single disc level alone, has contributed to a more comprehensive assessment of degeneration [3].

Atrophy and fatty infiltration of the multifidus and erector spinae muscles, which play an important role in lumbar spine stability, have been suggested to be associated with disc degeneration [4,5,6,7]. Many studies have found greater fatty infiltration and lower functional muscle area in the paraspinal muscles of patients with advanced disc degeneration [5,6,7,8,9]. However, some studies have reported that fatty infiltration, which reflects muscle quality, is a stronger marker than cross-sectional area measurements, which reflect muscle quantity [8,10]. Quantitative assessment of functional muscle area and fatty infiltration may therefore allow muscle quantity and quality to be evaluated together.

The underlying mechanism of the interaction between disc degeneration and paraspinal muscle changes remains unclear. While pain and biomechanical alterations associated with disc degeneration are thought to lead to muscle degeneration, it has also been suggested that structural changes in the paraspinal muscles may impair segmental stability and accelerate the degenerative process [11,12]. Therefore, the direction of causality in this relationship remains controversial.

Although a substantial proportion of studies on this subject have reported significant associations between disc degeneration and paraspinal muscle morphology, both disc degeneration and paraspinal muscle changes are strongly influenced by age, which may confound their observed relationship [6,13]. Previous MRI-based research in symptomatic patients has shown that sex, age, and intervertebral disc degeneration may each be independently associated with posterior paraspinal muscle fatty infiltration [14].

The primary aim of this study was to evaluate the associations between the Total Pfirrmann Score and quantitative measurements of relative functional cross-sectional area and fatty infiltration of the multifidus, erector spinae, and psoas muscles at L4–L5. The secondary aim was to examine the corresponding level-specific associations between the L4–L5 Pfirrmann grade and muscle morphology measured at the same level. In both analyses, we further evaluated whether the observed associations persisted after adjustment for age. We hypothesized that greater disc degeneration would be associated with lower functional muscle area and greater fatty infiltration, with attenuation of these associations after age adjustment.

## 2. Materials and Methods

### 2.1. Study Design and Patient Population

This retrospective cross-sectional study evaluated patients who underwent lumbar magnetic resonance imaging for low back pain between January 2023 and December 2024. Eligible patients were identified from the institutional imaging archive according to predefined inclusion and exclusion criteria. The inclusion criteria were defined as being between 20 and 55 years of age, having undergone lumbar MRI for low back pain, and having image quality suitable for quantitative muscle analysis. The exclusion criteria were defined as a history of previous lumbar surgery, spondylolisthesis, vertebral fracture, spinal tumor, infection, inflammatory spinal disease, congenital spinal anomalies, scoliosis (>10° Cobb angle), neuromuscular disorders, and inadequate image quality. The age range was restricted to reduce the confounding influence of advanced age-related changes on both lumbar disc degeneration and paraspinal muscle morphology.

The study protocol was approved by the Scientific Research Ethics Committee of Kanuni Sultan Süleyman Training and Research Hospital (Decision No: KAEK/2026.01.12, Date: 15 January 2026). The study was conducted in accordance with the principles of the Declaration of Helsinki. All data were anonymized before analysis. The requirement for informed consent was waived because of the retrospective design of the study and the use of anonymized patient data.

### 2.2. MRI Assessment

All MRI examinations were performed using a 1.5-Tesla magnetic resonance imaging system (Philips Ingenia, Philips Healthcare, Best, The Netherlands). All images were evaluated through the hospital PACS system.

Lumbar intervertebral disc degeneration was graded on sagittal T2-weighted images according to the Pfirrmann classification. The discs at the L1–L2, L2–L3, L3–L4, L4–L5, and L5–S1 levels were evaluated separately. To represent the total degenerative burden, the Pfirrmann grades of all levels were summed, and the Total Pfirrmann Score was calculated. The Total Pfirrmann Score ranged from 5 to 25, with higher values indicating greater cumulative lumbar disc degeneration. The L4–L5 Pfirrmann grade was additionally retained for level-specific analyses corresponding to the level of muscle measurement. Accordingly, the Total Pfirrmann Score and the L4–L5 Pfirrmann grade were analyzed as distinct measures representing cumulative whole-lumbar degeneration and local level-specific degeneration, respectively.

### 2.3. Paraspinal Muscle and Intervertebral Disc Area Analysis 

Quantitative measurements were performed on axial T2-weighted images obtained at the L4–L5 intervertebral disc level. Image analyses were performed using ImageJ software, version 1.54g (National Institutes of Health, Bethesda, MD, USA). The axial image passing through the center of the L4–L5 intervertebral disc was selected. This level provides clear visualization of the multifidus, erector spinae, and psoas muscles.

The multifidus (MF), erector spinae (ES), and psoas (PS) muscles were delineated bilaterally using manual segmentation. On the same axial slice, the L4–L5 intervertebral disc area was also manually outlined and measured. The observer performing the muscle measurements was blinded to the level-specific Pfirrmann grades and the Total Pfirrmann Score.

**Figure 1:**
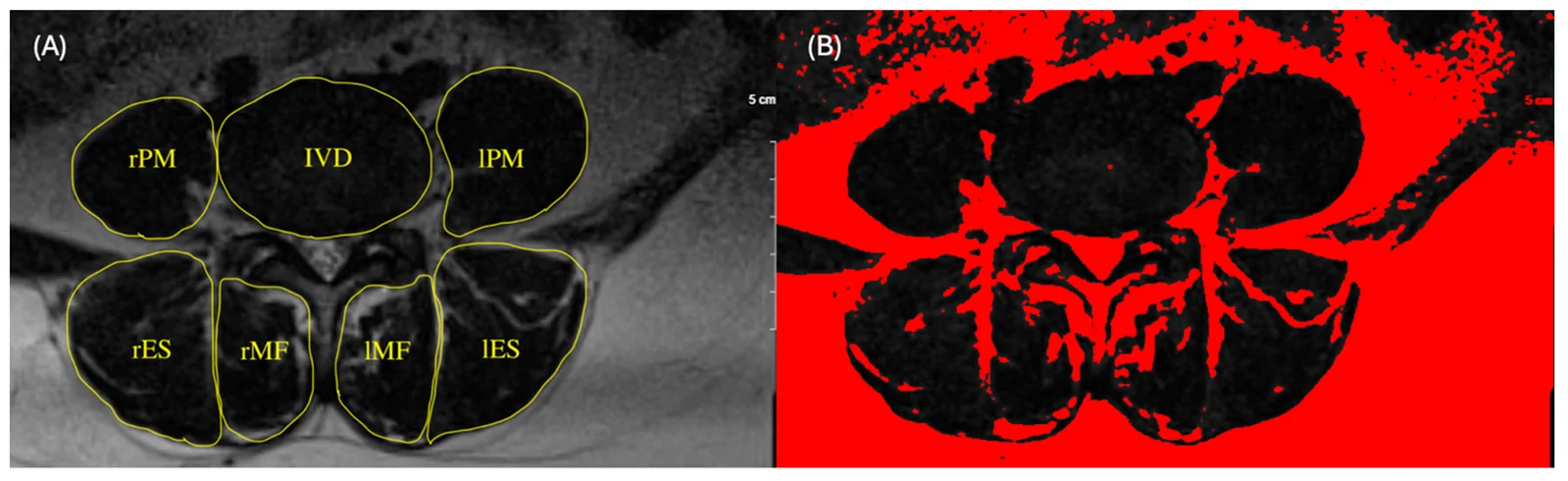
Representative axial T2-weighted lumbar magnetic resonance image demonstrating paraspinal muscle segmentation and fatty infiltration analysis. (**A**) Manual delineation of the bilateral psoas major (PM), erector spinae (ES), multifidus (MF), and intervertebral disc (IVD) regions of interest. (**B**) Threshold-based image segmentation used to identify fatty infiltration within the selected regions. Red pixels represent adipose tissue, whereas black pixels represent lean muscle tissue and other non-fat structures. r, right; l, left; PM, psoas major; ES, erector spinae; MF, multifidus; IVD, intervertebral disc.

For each muscle, total cross-sectional area (TCSA), functional cross-sectional area (FCSA), relative functional cross-sectional area (rFCSA), and percentage of fatty infiltration (FI%) were calculated. Functional cross-sectional area was defined as the remaining fat-free muscle tissue after exclusion of intramuscular adipose tissue using the thresholding method. For each patient, the grayscale threshold was individually adjusted according to the signal-intensity characteristics of the corresponding axial T2-weighted image to distinguish lean muscle tissue from intramuscular fat within the manually delineated region of interest. The same anatomical delineation and thresholding procedure was applied systematically to all images (Figure 1).

The percentage of fatty infiltration was calculated using the following formula:

FI (%) = [(TCSA − FCSA)/TCSA] × 100

To reduce the effects of body habitus and anatomical differences, relative functional cross-sectional area (rFCSA) was calculated by normalizing the functional muscle area to the intervertebral disc area at the same level:

rFCSA = FCSA/Intervertebral Disc Area

This same-level intervertebral disc area normalization approach has previously been used in quantitative MRI studies of paraspinal muscle morphology to reduce interindividual anatomical variability [15]. This provided an internal anatomical reference from the same axial image but did not substitute for direct adjustment for body mass index or body composition.

The mean values of the right- and left-sided measurements were used in the statistical analyses.

### 2.4. Reliability Analysis

All measurements were initially performed by an observer experienced in spinal imaging. For interobserver reliability assessment, images from 20 randomly selected patients were independently re-evaluated by a second observer who was blinded to the initial measurements. Continuous measurements were assessed using a two-way random-effects, single-measure intraclass correlation coefficient for absolute agreement [ICC(2, 1)] with 95% confidence intervals. Agreement for the level-specific ordinal Pfirrmann grades was evaluated using quadratic weighted kappa coefficients with percentile bootstrap 95% confidence intervals based on 5000 resamples.

Reliability estimates were interpreted as follows: ICC values below 0.50 indicated poor reliability, values from 0.50 to 0.75 indicated moderate reliability, values from 0.75 to 0.90 indicated good reliability, and values above 0.90 indicated excellent reliability.

### 2.5. Statistical Analysis

Statistical analyses were performed using IBM SPSS Statistics version 26.0 (IBM Corp., Armonk, NY, USA).

Continuous variables were presented as mean ± standard deviation, and categorical variables were presented as numbers and percentages. The normality of data distribution was assessed using the Kolmogorov–Smirnov test. Distributional characteristics were additionally evaluated using histograms and normal probability plots.

The relationships between the Total Pfirrmann Score and muscle parameters were examined using Spearman correlation analysis. Level-specific relationships between the L4–L5 Pfirrmann grade and muscle parameters measured at the same level were also evaluated using Spearman correlation analysis.

Patients were divided into three groups according to age (20–34 years, 35–44 years, and 45–55 years). Comparisons between groups were performed using one-way analysis of variance (ANOVA) or the Kruskal–Wallis test. When an overall group difference was statistically significant, Tukey’s honestly significant difference test was used after one-way ANOVA, whereas Dunn’s test with Bonferroni adjustment was used after the Kruskal–Wallis test.

Multiple linear regression analysis was performed with the Total Pfirrmann Score as the dependent variable because the primary analytical objective was to evaluate whether paraspinal muscle morphology was independently associated with the overall burden of lumbar disc degeneration. This model specification addressed an association-based research question and was not intended to imply a causal or temporal direction. Age, multifidus rFCSA, erector spinae rFCSA, multifidus fatty infiltration, and erector spinae fatty infiltration were included in the model. Because the Total Pfirrmann Score is the sum of five ordinal grades and spans a 21-point range (5–25), it was treated as an approximately continuous measure of cumulative degeneration. Linearity, residual normality, and homoscedasticity were evaluated using residual plots, normal probability plots, and the Breusch–Pagan test. Multicollinearity was assessed using tolerance and variance inflation factor values. Because heteroscedasticity was identified, HC3 heteroscedasticity-consistent robust standard errors were used for regression inference.

To evaluate the potential confounding effect of age, age-controlled partial correlation analyses were performed separately for cumulative and level-specific outcomes. For the Total Pfirrmann Score, age-controlled correlations were calculated for multifidus and erector spinae fatty infiltration. For the L4–L5 Pfirrmann grade, age-controlled correlations were calculated for multifidus rFCSA, erector spinae rFCSA, psoas rFCSA, multifidus FI, and erector spinae FI.

A sensitivity power analysis indicated that a sample of 84 patients provided 80% power, at a two-sided alpha level of 0.05, to detect a correlation coefficient of approximately |r| = 0.30 or greater.

A *p* value of <0.05 was considered statistically significant in all analyses. All statistical tests were two-sided.

## 3. Results

### 3.1. Patient Characteristics

A total of 84 patients who underwent lumbar MRI for low back pain were included in the study. Of these patients, 48 (57.1%) were female and 36 (42.9%) were male. The mean age was 42.3 ± 8.4 years (range, 20–55 years). The mean Total Pfirrmann Score was 14.3 ± 3.8. No significant difference was found between female and male patients in terms of Total Pfirrmann Scores (14.31 ± 3.93 vs. 14.35 ± 3.66, *p* = 0.954) (Table 1).

### 3.2. Interobserver Reliability

Interobserver reliability was assessed in 20 randomly selected patients by a second independent observer blinded to the initial measurements. For continuous measurements, ICC(2, 1) values ranged from 0.555 to 0.989, indicating moderate to excellent reliability. Quadratic weighted kappa values for level-specific Pfirrmann grades ranged from 0.859 to 0.947, indicating almost perfect agreement. The ICC for the Total Pfirrmann Score was 0.977 (95% CI, 0.959–0.985) (Table 2). Although psoas fatty infiltration was measured as part of the quantitative protocol, the right psoas fat ratio showed the lowest interobserver reliability (ICC = 0.555; 95% CI, 0.234–0.747), while the left psoas fat ratio showed moderate reliability (ICC = 0.725; 95% CI, 0.204–0.882). Therefore, psoas FI was not retained in the principal correlation or age-controlled analyses.

### 3.3. Relationship Between Age and Disc Degeneration

A significant positive correlation was found between age and the Total Pfirrmann Score (rho = 0.519, *p* < 0.001). In addition, significant differences were observed between age groups in terms of Total Pfirrmann Scores and multifidus fatty infiltration. Post hoc comparisons showed that the 45–55-year group had a higher Total Pfirrmann Score than both the 20–34-year group (*p* < 0.001) and the 35–44-year group (*p* = 0.016). Multifidus rFCSA was lower in the 45–55-year group than in both the 20–34-year group (*p* = 0.019) and the 35–44-year group (*p* = 0.019). Multifidus fatty infiltration was higher in the 45–55-year group than in the 20–34-year group (*p* = 0.026), whereas the remaining pairwise comparisons were not statistically significant (Table 3).

### 3.4. Relationship Between Total Pfirrmann Score and Paraspinal Muscle Parameters

A positive correlation was found between the Total Pfirrmann Score and multifidus fatty infiltration (rho = 0.316, *p* = 0.003). Similarly, a positive correlation was observed between erector spinae fatty infiltration and the Total Pfirrmann Score (rho = 0.300, *p* = 0.006). A negative correlation was found between psoas relative functional cross-sectional area and the Total Pfirrmann Score (rho = −0.247, *p* = 0.023). No statistically significant relationship was found between the relative functional cross-sectional areas of the multifidus and erector spinae muscles and the Total Pfirrmann Score (Table 4).

### 3.5. Degeneration and Muscle Parameters at the L4–L5 Level

A higher L4–L5 Pfirrmann grade was associated with lower multifidus rFCSA (rho = −0.233, *p* = 0.033) and lower psoas rFCSA (rho = −0.319, *p* = 0.003). The inverse association with erector spinae rFCSA was borderline (rho = −0.215, *p* = 0.050). A positive correlation was found between the L4–L5 Pfirrmann grade and erector spinae fatty infiltration (rho = 0.245, *p* = 0.025), whereas the association with multifidus fatty infiltration was not statistically significant (rho = 0.200, *p* = 0.069) (Table 5).

### 3.6. Multiple Linear Regression Analysis

Age, multifidus rFCSA, erector spinae rFCSA, multifidus fatty infiltration, and erector spinae fatty infiltration were included in the model, with the Total Pfirrmann Score as the dependent variable. The model explained 33.9% of the variance in the Total Pfirrmann Score (R^2^ = 0.339). Residuals were normally distributed (Shapiro–Wilk *p* = 0.620), and no evidence of nonlinearity was detected (RESET *p* = 0.749). Variance inflation factor values ranged from 1.32 to 1.42, indicating no relevant multicollinearity. Because heteroscedasticity was detected (Breusch–Pagan *p* = 0.002), HC3 robust standard errors were used. In the robust model, age was the only statistically significant predictor (B = 0.206, 95% CI, 0.105–0.307; *p* < 0.001) (Table 6).

### 3.7. Age-Controlled Partial Correlation Analysis

After controlling for age, the associations of the Total Pfirrmann Score with multifidus fatty infiltration (partial r = 0.193, *p* = 0.079) and erector spinae fatty infiltration (partial r = 0.194, *p* = 0.078) were no longer statistically significant.

In the age-controlled L4–L5 analysis, only the inverse association between the Pfirrmann grade and psoas rFCSA remained statistically significant (partial r = −0.232, *p* = 0.034). The associations with multifidus rFCSA (partial r = −0.110, *p* = 0.322), erector spinae rFCSA (partial r = −0.163, *p* = 0.141), multifidus FI (partial r = 0.060, *p* = 0.589), and erector spinae FI (partial r = 0.122, *p* = 0.271) were not statistically significant (Table 7).

## 4. Discussion

The most important finding of our study is that age was the only statistically significant predictor of the Total Pfirrmann Score in the multivariable model. Aging is known to cause structural changes in both intervertebral discs and paraspinal muscles [6]. Previous studies have shown that fatty infiltration of the paraspinal muscles increases with age, and that this change is particularly prominent in the multifidus and erector spinae muscles [6]. Similarly, the prevalence and severity of disc degeneration also increase with age [3,6]. The persistence of this association despite restricting the cohort to 20–55 years further highlights the importance of age even within a relatively compressed age range. Moreover, the associations between the Total Pfirrmann Score and posterior paraspinal muscle fatty infiltration were attenuated and did not reach statistical significance after adjustment for age, indicating that age may explain a substantial proportion of the observed muscle–disc relationships without excluding weaker independent associations.

The cumulative and level-specific analyses provided related but distinct information. Greater cumulative lumbar degeneration was associated with higher multifidus and erector spinae fatty infiltration and lower psoas rFCSA, whereas multifidus and erector spinae rFCSA were not significantly associated with the Total Pfirrmann Score. At L4–L5, higher Pfirrmann grade was associated with lower multifidus and psoas rFCSA, while the inverse association with erector spinae rFCSA was borderline. After adjustment for age, only the level-specific association between L4–L5 Pfirrmann grade and psoas rFCSA remained statistically significant.

The relationship between disc degeneration and paraspinal muscle degeneration has been extensively investigated in recent years. Previous studies have demonstrated that increased fatty infiltration of the multifidus and erector spinae muscles is associated with more advanced disc degeneration [3,4,5]. MRI-based studies conducted in large patient series have also reported significant associations between paraspinal muscle mass and lumbar disc degeneration, with this relationship being particularly pronounced in the posterior paraspinal muscles [4,5]. The positive correlations observed in our study between the total Pfirrmann score and fatty infiltration of the multifidus and erector spinae muscles are consistent with these findings. A closely comparable study in symptomatic patients reported that age, sex, and intervertebral disc degeneration were independently associated with posterior paraspinal muscle fatty infiltration [14]. In contrast, age was the only statistically significant predictor in our model, which may reflect differences in age range, sample characteristics, muscle normalization, and analytical approach.

In the assessment of paraspinal muscle quality, fatty infiltration has been suggested to be a more sensitive marker than cross-sectional area measurements, which mainly reflect muscle quantity [7,8]. Studies using quantitative MRI methods have shown that fat content in the multifidus and erector spinae muscles is associated with Pfirrmann grade and other degenerative spinal findings [7,8]. In addition, patients with multilevel disc degeneration have been reported to have higher levels of fatty infiltration than those with single-level degeneration [9]. In our study, the association between fatty infiltration of the multifidus and erector spinae muscles and the total Pfirrmann score supports the view that paraspinal muscle quality is related to the overall degenerative burden. A multicenter quantitative MRI study of 493 patients similarly found that increasing disc degeneration was associated more consistently with paraspinal muscle fat fraction than with muscle cross-sectional area; however, these associations were no longer significant after adjustment for age, sex, and BMI [16]. The attenuation observed in our age-controlled analyses is consistent with this pattern and indicates that the associations involving posterior muscle fatty infiltration should not be interpreted independently of age.

At the L4–L5 level, the associations with multifidus and psoas rFCSA suggest that local disc degeneration may be accompanied by reduced functional muscle area at the same segment. However, after adjustment for age, the multifidus and erector spinae associations were no longer statistically significant, whereas the inverse association with psoas rFCSA persisted. This finding may reflect differences between the psoas and posterior paraspinal muscles in anatomical location, loading pattern, and function. Nevertheless, the modest effect size and cross-sectional design preclude conclusions regarding the direction or mechanism of this association.

The use of a single lower-lumbar measurement level is supported by previous work showing that L4-level paraspinal muscle fatty infiltration correlates strongly with overall lumbar paraspinal muscle fat burden and is frequently used as a representative level [17]. A separate study also selected L4 because axial slices are consistently orthogonal to the muscle at this level, supporting reproducible quantitative assessment [18].

The Total Pfirrmann Score was used to represent cumulative lumbar degenerative burden, whereas the L4–L5-specific analysis assessed local concordance between disc degeneration and muscle morphology measured at the same level. Although these analyses provide complementary global and segmental information, comparing a single-level muscle measurement with a whole-lumbar score may attenuate or distort associations when degeneration is concentrated at other levels.

The mechanism underlying the relationship between disc degeneration and paraspinal muscle changes has not yet been fully clarified. Current views suggest that this relationship is not unidirectional, but rather represents a process of reciprocal interaction [11,12]. Pain, restricted mobility, and biomechanical changes caused by disc degeneration may lead to muscle atrophy and fatty infiltration, whereas structural changes in the paraspinal muscles may impair segmental stability and accelerate the degenerative process [11,12]. Recent longitudinal data have also shown that paraspinal muscle atrophy may be associated with the progression of vertebral endplate degeneration [13]. Although the Total Pfirrmann Score was specified as the dependent variable to address the study’s primary association-based question, the present cross-sectional findings cannot determine whether muscle changes precede disc degeneration, result from it, or develop in parallel through shared age-related mechanisms; therefore, the direction of the regression model should not be interpreted as evidence of causality or temporal sequence.

The age-controlled partial correlations for multifidus and erector spinae fatty infiltration remained positive but did not reach statistical significance. These findings should not be interpreted as proof that no residual association exists. The sensitivity analysis indicated that the study had 80% power to detect correlations of approximately |r| = 0.30 or greater; therefore, the observed age-adjusted correlations of approximately 0.19 may have been underpowered, and a larger sample could reveal a weak but statistically significant independent relationship. Although variance inflation factor values did not indicate problematic statistical multicollinearity, age affects both disc degeneration and muscle morphology, and may account for a substantial proportion of their shared biological variance.

The Total Pfirrmann Score was treated as an approximately continuous cumulative measure because it combines five ordinal grades across a 21-point range. The regression assumptions were acceptable, and HC3 robust standard errors were used because heteroscedasticity was detected. Age remained the only statistically significant predictor under this approach; nevertheless, the ordinal origin of the summed score should be considered when interpreting the regression findings.

Psoas fatty infiltration was measured as part of the quantitative protocol; however, its interobserver reliability was lower than that of the other retained measurements, particularly on the right side. Therefore, psoas FI was not retained in the principal correlation or age-controlled analyses and was not included in the main interpretation. In contrast, psoas area measurements demonstrated excellent reliability, supporting the interpretation of the rFCSA findings.

This study has several limitations. First, it has a retrospective and cross-sectional design; therefore, a causal relationship cannot be established. Second, muscle measurements were evaluated only at the L4–L5 level, and changes at other levels were not examined. The study was also conducted at a single center in a symptomatic population, which may limit generalizability. Potential confounding factors, including physical activity, occupational loading, symptom duration, and muscle strength, were unavailable. BMI data were not available in the retrospective dataset and therefore could not be included in the multivariable analysis. This represents a major limitation, particularly for the interpretation of muscle fatty infiltration, because systemic adiposity and related metabolic factors may independently affect intramuscular fat accumulation. Normalization of FCSA to the corresponding L4–L5 disc area reduced variation related to anatomical size but did not account for systemic adiposity or substitute for BMI or direct body-composition assessment, and disc area itself may vary with degeneration and spinal level. Consequently, residual confounding related to body composition cannot be excluded, and the clinical generalizability of the regression model may be limited in populations with different BMI or adiposity distributions. The individualized thresholding procedure was applied systematically, but image-dependent threshold selection may still introduce operator variability. Restricting the cohort to 20–55 years also limits generalizability to older populations. Strengths of the study include the quantitative evaluation of muscle quantity and quality, blinded segmentation, interobserver reliability testing, and the separate assessment of cumulative and L4–L5-specific degeneration with age-controlled analyses.

## 5. Conclusions

Cumulative lumbar disc degeneration was associated with greater multifidus and erector spinae fatty infiltration and lower psoas rFCSA, whereas multifidus and erector spinae rFCSA were not associated with the Total Pfirrmann Score. Age was the only statistically significant predictor of the Total Pfirrmann Score in the multivariable model. At L4–L5, only the inverse association between Pfirrmann grade and psoas rFCSA remained statistically significant after adjustment for age. These findings indicate that age accounts for a substantial proportion of the observed associations between posterior paraspinal muscle morphology and lumbar disc degeneration; however, weak independent associations cannot be excluded. Prospective longitudinal studies are needed to clarify the temporal relationship between disc degeneration and muscle changes.

## Figures and Tables

**Table 1:** Baseline characteristics of the study population.

Variable	Value
Number of patients	84
Age (years)	42.3 ± 8.4
Age range (years)	20–55
Female, *n* (%)	48 (57.1)
Male, *n* (%)	36 (42.9)
Total Pfirrmann Score	14.3 ± 3.8
Male Total Pfirrmann Score	14.31 ± 3.93
Female Total Pfirrmann Score	14.35 ± 3.66
Sex comparison, *p* value	0.954

Data are presented as mean ± standard deviation or *n* (%), as appropriate. *n*, number.

**Table 2:** Interobserver reliability of Pfirrmann grading and quantitative MRI measurements.

Measurement	Statistic	Estimate	95% CI
L1–L2 Pfirrmann grade	Quadratic weighted kappa	0.898	0.745–1.000
L2–L3 Pfirrmann grade	Quadratic weighted kappa	0.864	0.676–1.000
L3–L4 Pfirrmann grade	Quadratic weighted kappa	0.940	0.854–1.000
L4–L5 Pfirrmann grade	Quadratic weighted kappa	0.859	0.705–0.947
L5–S1 Pfirrmann grade	Quadratic weighted kappa	0.947	0.826–1.000
Total Pfirrmann Score	ICC(2, 1)	0.977	0.959–0.985
L4–L5 intervertebral disc area	ICC(2, 1)	0.974	0.929–0.988
Right multifidus muscle area	ICC(2, 1)	0.902	0.779–0.948
Right multifidus fat ratio	ICC(2, 1)	0.870	0.703–0.941
Right erector spinae muscle area	ICC(2, 1)	0.942	0.847–0.980
Right erector spinae fat ratio	ICC(2, 1)	0.795	0.373–0.925
Right psoas major muscle area	ICC(2, 1)	0.989	0.968–0.995
Right psoas major fat ratio	ICC(2, 1)	0.555	0.234–0.747
Left multifidus muscle area	ICC(2, 1)	0.825	0.539–0.929
Left multifidus fat ratio	ICC(2, 1)	0.888	0.701–0.964
Left erector spinae muscle area	ICC(2, 1)	0.908	0.786–0.967
Left erector spinae fat ratio	ICC(2, 1)	0.795	0.238–0.889
Left psoas major muscle area	ICC(2, 1)	0.988	0.964–0.995
Left psoas major fat ratio	ICC(2, 1)	0.725	0.204–0.882

All analyses were based on 20 randomly selected patients. CI, confidence interval; ICC, intraclass correlation coefficient; kappa, kappa.

**Table 3:** Association of age with disc degeneration and ultifidus fatty infiltration.

Variable	20–34 Years (*n* = 19)	35–44 Years (*n* = 22)	45–55 Years (*n* = 43)	*p* Value	Significant Post Hoc Comparison
Total Pfirrmann Score	11.58 ± 2.99	13.50 ± 2.54	15.98 ± 3.78	<0.001	45–55 vs. 20–34: *p* < 0.001; 45–55 vs. 35–44: *p* = 0.016
Multifidus rFCSA	0.602 ± 0.116	0.598 ± 0.099	0.524 ± 0.099	0.004	45–55 vs. 20–34: *p* = 0.019; 45–55 vs. 35–44: *p* = 0.019
Multifidus FI (%)	15.78 ± 5.54	16.85 ± 4.60	20.26 ± 7.00	0.015	45–55 vs. 20–34: *p* = 0.026

Data are presented as mean ± standard deviation. Overall comparisons were performed using one-way analysis of variance, followed by Tukey’s honestly significant difference test. FI, fatty infiltration; rFCSA, relative functional cross-sectional area.

**Table 4:** Correlations between total Pfirrmann score and paraspinal muscle parameters.

Variable	Spearman ρ	*p* Value
Multifidus rFCSA	−0.184	0.094
Erector spinae rFCSA	−0.184	0.095
Psoas rFCSA	−0.247	0.023
Multifidus FI (%)	0.316	0.003
Erector spinae FI (%)	0.300	0.006

FI, fatty infiltration; rFCSA, relative functional cross-sectional area.

**Table 5:** Correlations between L4–L5 Pfirrmann grade and muscle parameters measured at the same level.

Variable	Spearman ρ	*p* Value
Multifidus rFCSA	−0.233	0.033
Erector spinae rFCSA	−0.215	0.050
Psoas rFCSA	−0.319	0.003
Multifidus FI (%)	0.200	0.069
Erector spinae FI (%)	0.245	0.025

FI, fatty infiltration; rFCSA, relative functional cross-sectional area.

**Table 6:** Multiple linear regression analysis for predictors of total Pfirrmann score using HC3 robust standard errors.

Predictor	B	Robust SE	95% CI	*p* Value
Age	0.206	0.051	0.105–0.307	<0.001
Multifidus rFCSA	0.979	3.885	−6.756–8.714	0.802
Erector spinae rFCSA	−1.995	2.648	−7.267–3.277	0.454
Multifidus FI (%)	0.086	0.068	−0.049–0.221	0.209
Erector spinae FI (%)	0.041	0.073	−0.104–0.185	0.576

Model R^2^ = 0.339. B, unstandardized regression coefficient; CI, confidence interval; FI, fatty infiltration; rFCSA, relative functional cross-sectional area; SE, standard error.

**Table 7:** Age-controlled partial correlation analyses.

Outcome	Muscle Parameter	Partial r	*p* Value
Total Pfirrmann Score	Multifidus FI (%)	0.193	0.079
Total Pfirrmann Score	Erector spinae FI (%)	0.194	0.078
L4–L5 Pfirrmann grade	Multifidus rFCSA	−0.110	0.322
L4–L5 Pfirrmann grade	Erector spinae rFCSA	−0.163	0.141
L4–L5 Pfirrmann grade	Psoas rFCSA	−0.232	0.034
L4–L5 Pfirrmann grade	Multifidus FI (%)	0.060	0.589
L4–L5 Pfirrmann grade	Erector spinae FI (%)	0.122	0.271

FI, fatty infiltration; rFCSA, relative functional cross-sectional area. Partial correlations were controlled for age.

## Data Availability

The data supporting the findings of this study are available from the corresponding author upon reasonable request. The data are not publicly available because of patient privacy and ethical restrictions.

## Abbreviations

CSA	Cross-Sectional Area
FCSA	Functional Cross-Sectional Area
FI	Fatty Infiltration
ICC	Intraclass Correlation Coefficient
IVDD	Intervertebral Disc Degeneration
MRI	Magnetic Resonance Imaging
ROI	Region of Interest
SD	Standard Deviation
